# Supplementary material for: Role of helical edge modes in the chiral quantum anomalous Hall state
Source: Sci Rep. 2018 Jan 22;8:1335. doi: 10.1038/s41598-018-19272-7 (PMC5778147; doi:10.1038/s41598-018-19272-7)
Supplement: Supplementary file 1 — Supplementary Information [file 41598_2018_19272_MOESM1_ESM.pdf]

Supplementary Information

Role of helical edge modes in the chiral quantum anomalous Hall state

Arjun Mani and Colin Benjamin*

School of Physical Sciences, National Institute of Science Education & Research, HBNI, Jatni-752050, India

I. RELATION BETWEEN EQUILIBRATED POTENTIALS AND PROBE POTENTIALS

A. Relation between V_i' and V_i for Four terminal QAH bar with chiral(trivial) QAH edge mode and trivial QSH edge modes

See Section 2.3.2 and Fig. 4(b) of the manuscript for the details of the set-up. See Eq. 16 of the manuscript for relations between currents and voltages.

$$
\begin{aligned}
&(2-f-f_0)V_1 + \tfrac{[1-(f+f_0)][1+(f+f_0)R_2]T_2}{[1-R_2^2(f^2+f_0^2)]}V_2 + \tfrac{R_2(1-f-f_0)^2}{[1-R_2^2(f^2+f_0^2)]}V_2' = [(2-f-f_0) + \tfrac{[1-(f+f_0)][1+(f+f_0)R_2]T_2}{[1-R_2^2(f^2+f_0^2)]} + \tfrac{R_2(1-f-f_0)^2}{[1-R_2^2(f^2+f_0^2)]}]V_1',\\
&\tfrac{[2-(f+f_0)+((1-f)f+(1-f_0)f_0)R_2-(f-f_0)^2R_2^2)T_2}{[1-R_2^2(f^2+f_0^2)]}V_2 + [1-(f+f_0)]V_3 + \tfrac{(2+(-2+f)f+(-2+f_0)f_0)R_2+(f-f_0)^2R_2^3}{[1-R_2^2(f^2+f_0^2)]}V_1' =\\
&[\tfrac{[2-(f+f_0)+((1-f)f+(1-f_0)f_0)R_2-(f-f_0)^2R_2^2)T_2}{[1-R_2^2(f^2+f_0^2)]} + [1-(f+f_0)] + \tfrac{(-2+f)f+(-2+f_0)f_0)R_2+(f-f_0)^2R_2^3}{[1-R_2^2(f^2+f_0^2)]}]V_2',\\
&(2-f-f_0)V_3 + \tfrac{[1-(f+f_0)][1+(f+f_0)R_4]T_4}{[1-R_4^2(f^2+f_0^2)]}V_4 + \tfrac{R_2(1-f-f_0)^2}{[1-R_2^2(f^2+f_0^2)]}V_4' = [(2-f-f_0) + \tfrac{[1-(f+f_0)][1+(f+f_0)R_2]T_2}{[1-R_2^2(f^2+f_0^2)]} + \tfrac{R_2(1-f-f_0)^2}{[1-R_2^2(f^2+f_0^2)]}]V_3',\\
&\tfrac{[2-(f+f_0)+((1-f)f+(1-f_0)f_0)R_4-(f-f_0)^2R_4^2)T_4}{[1-R_4^2(f^2+f_0^2)]}V_4 + [1-(f+f_0)]V_1 + \tfrac{(2+(-2+f)f+(-2+f_0)f_0)R_4+(f-f_0)^2R_4^3}{[1-R_4^2(f^2+f_0^2)]}V_3' =\\
&[\tfrac{[2-(f+f_0)+((1-f)f+(1-f_0)f_0)R_4-(f-f_0)^2R_4^2)T_4}{[1-R_4^2(f^2+f_0^2)]} + [1-(f+f_0)] + \tfrac{(-2+f)f+(-2+f_0)f_0)R_4+(f-f_0)^2R_4^3}{[1-R_2^2(f^2+f_0^2)]}]V_4'.
\end{aligned}
\tag{1}
$$

B. Relation between V_i' and V_i for six terminal QAH bar with chiral(topological) QAH edge mode and trivial QSH edge modes

See Section 3.2.2 and Fig. 7(c) of the manuscript for the details of the set-up. See Eq. 22 of the manuscript for relations between currents and voltages.

$$
\begin{aligned}
&(1-f)(V_2+V_3) = 2(1-f)V_2', \quad (1-f)(V_5+V_6) = 2(1-f)V_5',\\
&(1-f)V_3 + (\tfrac{T_4(1-f)}{(1-R_4^2f^2)} + \tfrac{T_4R_4f(1-f)}{1-R_4^2f^2})V_4 + \tfrac{R_4(1-f)^2}{(1-R_4^2f^2)}V_4' = ((1-f) + (\tfrac{T_4(1-f)}{(1-R_4^2f^2)} + \tfrac{T_4R_4f(1-f)}{1-R_4^2f^2}) + \tfrac{R_4(1-f)^2}{(1-R_4^2f^2)})V_3',\\
&(1-f)V_6 + (\tfrac{T_1(1-f)}{(1-R_1^2f^2)} + \tfrac{T_1R_1f(1-f)}{1-R_1^2f^2})V_1 + \tfrac{R_1(1-f)^2}{(1-R_1^2f^2)}V_1' = ((1-f) + (\tfrac{T_1(1-f)}{(1-R_1^2f^2)} + \tfrac{T_1R_1f(1-f)}{1-R_1^2f^2}) + \tfrac{R_1(1-f)^2}{(1-R_1^2f^2)})V_3',\\
&(1-f)V_5 + (\tfrac{T_4(1-f)}{(1-R_4^2f^2)} + \tfrac{T_4R_4f(1-f)}{1-R_4^2f^2})V_4 + \tfrac{R_4(1-f)^2}{(1-R_4^2f^2)}V_3' = ((1-f) + (\tfrac{T_4(1-f)}{(1-R_4^2f^2)} + \tfrac{T_4R_4f(1-f)}{1-R_4^2f^2}) + \tfrac{R_4(1-f)^2}{(1-R_4^2f^2)})V_3',\\
&(1-f)V_2 + (\tfrac{T_1(1-f)}{(1-R_1^2f^2)} + \tfrac{T_1R_1f(1-f)}{1-R_1^2f^2})V_1 + \tfrac{R_1(1-f)^2}{(1-R_1^2f^2)}V_6' = ((1-f) + (\tfrac{T_1(1-f)}{(1-R_1^2f^2)} + \tfrac{T_1R_4f(1-f)}{1-R_1^2f^2}) + \tfrac{R_1(1-f)^2}{(1-R_1^2f^2)})V_3'.
\end{aligned}
\tag{2}
$$

with $a = 1 - R_2^2f^2, c = 1 - R_4^2f^2$.

C. Relation between V_i' and V_i for six terminal QAH bar with chiral(trivial) QAH edge mode and trivial QSH edge modes

See Section 3.3.2 and Fig. 7(d) of the manuscript for the details of the set-up. See Eq. 26 of the manuscript for relations between currents and voltages.

$$
\begin{aligned}
&(T_1(2-f-f_0)+T_1R_1(f(1-f)+f_0(1-f_0))/a+T_1R_1^2(f+f_0)(f(1-f)+f_0(1-f_0))/a)V_1+(1-f-f_0)V_2\\
&+(R_1((1-f)^2+(1-f_0)^2)+R_1^3((1-f)f+f_0(1-f_0))^2/a)V_6'=(T_1(2-f-f_0)+T_1R_1(f(1-f)+f_0(1-f_0))/a\\
&+T_1R_1^2(f+f0)(f(1-f)+f_0(1-f_0))/a+(1-f-f_0)+R_1((1-f)^2+(1-f_0)^2)+R_1^3((1-f)f+f_0(1-f_0))^2/a)V_1',\\
&(2-f-f_0)V_6+(T_1(1-f-f_0)/a+T_1R_1(f+f_0)(1-f-f_0)/a)V_1+R_1(1-f-f_0)^2/aV_1'=((2-f-f_0)\\
&+(T_1(1-f-f_0)/a+T_1R_1(f+f_0)\quad(1-f-f_0)/a)+R_1(1-f-f_0)^2/a)V_6',\\
&(2-f-f_0)V_2+(1-f-f_0)V_3=(3-2(f+f_0))V_2',\\
&(2-f-f_0)V_5+(1-f-f_0)V_6=(3-2(f+f_0))V_5',\\
&(T4(2-f-f_0)+T_4R_4(f(1-f)+f_0(1-f_0))/c+T_4R_4^2(f+f_0)(f(1-f)+f_0(1-f_0))/c)V_4+(1-f-f_0)V_5\\
&+(R_4((1-f)^2+(1-f_0)^2)+R_4^3((1-f)f+f_0(1-f_0))^2/c)V_3'=(T_4(2-f-f_0)+T_4R_4(f(1-f)+f_0(1-f_0))/c\\
&+T_4R_4^2(f+f_0)(f(1-f)+f_0(1-f_0))/c+(1-f-f_0)+R_4((1-f)^2+(1-f_0)^2)+R_4^3((1-f)f+f_0(1-f_0))^2\\
&(2-f-f_0)V3+(T_4(1-f-f_0)/c+T_4R_4(f+f_0)(1-f-f_0)/c)V_4+R_4(1-f-f_0)^2/cV_4'=((2-f-f_0)\\
&+(T_4(1-f-f_0)/c+T_4R_4(f+f_0)\quad(1-f-f_0)/c)+R_4(1-f-f_0)^2/c)V_3'
\end{aligned}
$$
